# Leptospirosis complicated with Guillain Barre syndrome, papillitis and thrombotic thrombocytopenic Purpura; a case report

**DOI:** 10.1186/s12879-018-3616-5

**Published:** 2018-12-22

**Authors:** Kavinga Kalhari Kobawaka Gamage, Harshini Fernando

**Affiliations:** National Hospital of Sri Lanka, Colombo 10, Sri Lanka

**Keywords:** Leptospirosis, Guillain Barre syndrome, Thrombotic thrombocytopenic Purpura, Papillitis

## Abstract

**Background:**

Leptospirosis is a zoonosis commonly prevalent in tropical countries. Clinical course of leptospirosis varies from mild to severe disease. Here we present a case of leptospirosis complicated with Guillain-Barre Syndrome (GBS), papillitis, and Thrombotic Thrombocytopenic Purpura(TTP).

**Case presentation:**

A 21-year-old Asian male presented with fever, myalgia, oliguria and dyspnoea where he was managed as for leptospirosis complicated with pulmonary haemorrhages and acute renal failure. Leptospirosis was confirmed by Microscopic Agglutination Test(MAT) with a fourfold rise in antibody titre between acute and convalescent serum. The highest antibody titre was against Leptospira antigen serogroup Semaranga (strain Patoc) (1:1280) followed by serogroup Australis (strain Australis) (1:640) and serogroup Autumnalis (strain Bankgkinang) (1:320). Two weeks later he developed blindness, ascending weakness of lower limbs with global areflexia and an acute inflammatory demyelinating polyradiculopathy(AIDP) variant GBS was confirmed with nerve conduction studies. TTP complicated the picture several days later. He was initiated on plasmapheresis where clinical improvement was seen after 14 cycles. He had an incomplete neurological recovery with permanent vision loss but completely recovered from TTP. He also had permanent renal impairment.

**Conclusion:**

Leptospirosis should be suspected and treated empirically in the relevant clinical settings where it can present with an atypical clinical picture as in our case with an acute febrile illness followed by GBS as well as TTP.

## Background

Leptospirosis is a zoonosis which is prevalent in both tropical and temperate regions being more common in tropics [1] as the pathogenic bacteria survives longer in such environment. Clinical manifestations are caused by the obligatory aerobic bacteria of the genus *Leptospira* which are spirochetes*.* Many mammalian species act as the natural host where the *Leptospira* lives in their kidneys [1]. Rodents are the most common reservoir in transmission of the disease and humans are infected incidentally after being exposed to infected animal tissue or excreta. Incubation period lasts for about 10 days.

Clinical course of the disease ranges from mild to life threatening severe disease and the presentation may vary. Weil’s disease, is a severe form of leptospirosis is complicated by jaundice, renal failure and multiorgan dysfunction. Pulmonary haemorrhages, acute respiratory distress syndrome, uveitis, optic neuritis, peripheral neuropathy, myocarditis and rhabdomyolysis are well known to occur with severe forms of leptospirosis [2–6].

Leptospirosis is associated with various neurological manifestations out of which aseptic meningitis being the commonest. Other neurological presentations include myeloradiculopathy, myelopathy, cerebellar dysfunction and rarely GBS [7]. TTP is another rare manifestation secondary to leptospirosis which is characterized by the pentad of fever, renal failure, neurological involvement, microangiopathic anaemia and thrombocytopenia.

Here we report the case report of a patient with leptospirosis complicated with both GBS, TTP and papillitis where their occurrence together in leptospirosis is very rare.

## Case presentation

A previously healthy 21 year old Asian male working as a waiter in a tourist hotel presented with fever, arthalgia, myalgia and progressively reducing urine output over four days. He developed shortness of breath with non-productive cough following hospital admission and was electively intubated due to respiratory failure. There was no obvious history of leptospirosis exposure. He had no significant past medical or surgical history. He was not on any long term medication and with the onset of fever had self-medicated with paracetamol but in correct dose and correct time intervals. There was no significant family history of neurological disease or lung disease. He is a non-alcoholic, non-smoker and does not abuse illicit drugs. He denied any high risk sexual behaviour.

On examination he was febrile, tachypnoeic and pale. There was icterus with conjunctival injection. He was haemodynamically stable. Lungs had bilateral diffuse coarse crepitations and was desaturating on air prior to intubation. Examination of the abdomen was unremarkable. Neurological examination was normal at this point.

Arterial blood gas analysis showed evidence of type two respiratory failure with mixed respiratory and metabolic acidosis. Chest x-ray showed bilateral diffuse pulmonary shadowing and high resolution computed tomography(HRCT) of chest showed features suggestive of pulmonary haemorrhages. With the suggestive clinical picture, even with the absence of exposure to leptospirosis he was started on intravenous ceftriaxone empirically along with high dose intravenous methyl prednisolone pulses (500 mg daily for 3 days) for the treatment of pulmonary haemorrhages. Initial full blood count had neutrophil leucocytosis (white blood cells 16,000/uL, neutrophils 85%, lymphocytes 12%) with thrombocytopenia (platelet count 98,000/uL). Haemoglobin was normal (13.5 g/dL). Initial urine full report had 45–50 pus cells and 2–3 red cells per high power field. There was sub nephrotic range proteinuria (urine protein to creatinine ratio 2.5 g/mmol). Ultrasound scan showed acute renal parenchymal changes. Serum creatinine was high ranging from 256 umol/L and 768 umol/L) where regular haemodialysis was initiated.

After two weeks of the onset of the illness while he was being weaned off from the ventilatory support with improvement of pulmonary haemorrhages, he developed sudden severe lower limb weakness followed by upper limb weakness over one day. There was no diplopia. Assessment of dysphagia and bladder involvement was difficult at that point due to the indwelling nasogastric tube and the urinary catheter. Limb weakness then progressed to respiratory muscle weakness and required continued ventilatory support.

Examination at this point revealed flaccid weakness of both upper and lower limbs with global areflexia. There was no sensory impairment. Pupils were dilated with sluggish pupillary response and there was disc swelling bilaterally on examination of the optic fundi. Following recovery patient had permanent visual impairment (visual acuity 6/60 bilaterally) with pale optic discs. Other cranial nerves were normal.

With the development of neurological symptoms nerve conduction studies were performed which showed evidence of sensory motor demyelinating type polyneuropathy suggestive of AIDP type GBS. Cerebrospinal fluid analysis done on the 10th day from the onset of neurological symptoms showed cyto-protein dissociation. He was started on intravenous immunoglobulin 0.4 g/kg/day.

By day 18 of the illness there was progressive thrombocytopenia (lowest platelet count 15,000/uL) and severe anaemia (haemoglobin 5.6 g/dL). His lactate dehydrogenase (LDH 950 U/L) and indirect bilirubin was high with blood picture evidence of microangiopathic haemolytic anaemia. Direct antiglobulin test was negative. Prothrombin time and activated partial thromboplastin time was within the normal range throughout the hospital stay. Diagnosis of TTP was made and plasmapheresis was initiated as it would treat both TTP and GBS. Human immunodeficiency virus antibodies, the Venereal Disease Research Laboratory test, Mycoplasma antibodies, Epstein Barr and Cytomegalovirus antibodies were negative. Magnetic Resonance Imaging (MRI) brain with Magnetic Resonance Angiogram (MRA), and Magnetic Resonance Venogram (MRV) brain was normal. Renal biopsy showed focal glomerular necrosis and acute tubular injury together with some evidence of infection. A 15 panel leptospirosis Microscopic Agglutination Test (MAT) done on day 8 of the illness revealed a high titre for leptospira antigen serogroup Semaranga (strain Patoc) (1:1280) followed by serogroup Australis (strain Australis) (1:640) and serogroup Autumnalis (strain Bankgkinang) (1:320) while serovars *bataviae, bakeri, ratnapura, hardjo, icterohaemorrhagiae, pyrogenes, pomona, hebdomadid, cynopteri, canicola, javanka* and *Sarmin* had insignificant titres (< 1:20). In the convalescent phase MAT titres had increased to a four fold rise for serovars *patoc, australis* and *bangkinang* diagnosing leptospirosis.

Intravenous Ceftriaxone was continued for 14 days. Neurological improvement was noted following 14 cycles of plasmapheresis and he recovered from TTP by 12 cycles of plasmapheresis. But complete neurological recovery was not achieved at the end of the hospital stay and he was planned for long term neurological rehabilitation and he ended with permanent bilateral visual loss. He was dialysis dependent on discharge from the hospital due to progression to chronic kidney disease.

## Discussion and conclusions

Leptospirosis is a commonly prevailing infection in Sri Lanka [8] and is mostly prevalent in the farming community. Even though our patient did not reveal leptospirosis exposure, he was started on treatment with the typical initial clinical picture. He suffered from severe leptospirosis infection complicated with GBS, TTP and papillitis and outcome was poor due to the incomplete resolution of neurological complications.

Leptospirosis MAT needs the illness to last at least 8 days to be performed. MAT is considered the reference immunological test, and detects both immunoglobulin M (IgM) and immunoglobulin G class agglutinating antibodies [9]. MAT has a lower sensitivity in the acute phase but has higher specificity. Therefore, diagnosis is done on clinical grounds and treatment should be initiated empirically without waiting for confirmation of the diagnosis. IgM enzyme-linked immunosorbent assay (ELISA) is another diagnostic test which can be used in the acute setting of the illness and has lower specificity than MAT in the acute phase [9, 10].

GBS is well known to be associated with Campylobacter infection, and the pathogenesis is extensively studied. Occurrence of GBS is high, sometimes 77 times higher in those who had Campylobacter infection compared to the general population [11]. It is also known to be associated with other infections like influenza like illnesses, Cytomegalovirus, Epstein- Barr virus. But mechanism that Leptospirosis gives rise to GBS and its management is under evaluated probably due to the rare occurrence. It is likely through an immune mediated mechanism that leptospira causes GBS.

R. W. Ross Russel [12] from Malaya reports the first case of GBS associated with leptospirosis in 1956 where he reports it as an ascending myelitis with generalized areflexia and cyto-protein dissociation. Our patient had a severe form of AIDP variant of GBS and had possible residual axonal damage and had incomplete recovery despite 14 cycles of plasma exchange. Several studies have noted that rapid involvement of the respiratory muscles requiring ventilator support within 24 h of the illness, severe muscle weakness with grade 0 power in all four limbs, presence of cardiovascular autonomic symptoms, and electrophysiology showing unexcitable peripheral nerves [13–15] as markers of poor prognosis in patients with GBS. These factors may have played a role in the poor outcome of our patient as the other few documented cases of GBS associated with leptospirosis had showed good outcome with complete neurological recovery [16, 17]. Bal et al. reports a case of GBS in a paediatric patient following leptospirosis where she had made a complete recovery [17].

Ocular involvement in leptospirosis is known to occur in the systemic bacteraemic phase and the immunologic phase. Leptospirosis can present as subconjunctival haemorrhages, uveitis, papillitis, retinal vasculitis and retinal haemorrhages [4, 18]. Patient may be asymptomatic in most instances and these complications should be actively looked in to and the manifestations can present from early stage of infection to even upto 18 months later. Optic neuritis is noted as a common ocular manifestations in a study by Mancel E et al. in New Caledonia where the final outcome was very severe in 35% out of the 13 patients studied [19].

Leptospirosis and its association with TTP was first described in 1990 by Lain et al [20] where the patient had a fatal outcome. Following that several case reports have reported the association. In some cases, it has been occurring as an isolated condition in leptospirosis [21]. The freely unavailability of A Disintegrin and Metalloproteinase with Thrombospondin motifs 13 (ADAMTS13) protein measurements in such circumstances is a disadvantage in developing countries as leptospirosis per se will give rise to acute renal impairment but the whole clinical picture with the presence of fever, microangiopathic haemolytic anaemia with renal and neurological complications are helpful in diagnosis.

In conclusion leptospirosis should be suspected in endemic areas as a cause for atypical presentations of neurological symptoms or TTP following a febrile illness.

## Abbreviations

ADAMTS13: A Disintegrin and Metalloproteinase with Thrombospondin motifs
AIDP: Acute Inflammatory Demyelinating Polyradiculopathy
ELISA: enzyme-linked immunosorbent assay
GBS: Guillain Barre Syndrome
HRCT: High Resolution Computed Tomography
IgM: Immunoglobulin M
MAT: Microscopic Agglutination Test
MRA: Magnetic Resonance Angiogram
MRI: Magnetic Resonance Imaging
MRV: Magnetic Resonance Venogram
TTP: Thrombotic Thombocytopenic Purpura

## References

[1] Hartskeerl RA, Collares-Pereira M, Ellis WA (2011). Emergence, control and re-emerging leptospirosis: dynamics of infection in the changing world. Clin Microbiol Infect.

[2] Togal T (2010). Intensive care of a weils disease with multiorgan failure. J Clin Med Res.

[3] Cetin BD (2004). Acute renal failure: a common manifestation of leptospirosis. Ren Fail.

[4] Rathinam SR (2005). Ocular manifestations of leptospirosis. J Postgrad Med.

[5] Levett PN (2001). Leptospirosis. Clin Microbiol Rev.

[6] Rathinam SR (1997). Uveitis associated with an epidemic outbreak of leptospirosis. Am J Ophthalmol.

[7] Panicker JN, Mammachan R, Jayakumar RV (2001). Primary neuroleptospirosis. Postgrad Med J.

[8] Warnasekara JNA (2017). S., *Leptospirosis in Sri Lanka*. Sri Lankan Journal of Infectious Diseases.

[9] Niloofa R (2015). Diagnosis of leptospirosis: comparison between microscopic agglutination test, IgM-ELISA and IgM rapid Immunochromatography test. PLoS One.

[10] Panwala T, Rajdev S, Mulla S (2015). To evaluate the different rapid screening tests for diagnosis of leptospirosis. J Clin Diagn Res.

[11] Tam CC (2006). Incidence of Guillain-Barré syndrome among patients with campylobacter infection: a general practice research database study. J Infect Dis.

[12] Russell RW (1959). Neurological aspects of leptospirosis. J Neurol Neurosurg Psychiatry.

[13] Nithyashree N (2014). Factors predicting poor outcome in patients with fulminant Guillaine-Barre syndrome. Ann Indian Acad Neurol.

[14] Walgaard C (2011). Early recognition of poor prognosis in Guillain-Barre syndrome. Neurology.

[15] van Koningsveld R (2007). A clinical prognostic scoring system for Guillain-Barre syndrome. Lancet Neurol.

[16] Silva AP (2011). Leptospirosis presenting as ascending progressive leg weakness and complicating with acute pancreatitis. Braz J Infect Dis.

[17] Bal AM (2003). Guillain-Barre syndrome in a pediatric patient following infection due to Leptospira. Jpn J Infect Dis.

[18] Martins MG (1998). Ocular manifestations in the acute phase of leptospirosis. Ocul Immunol Inflamm.

[19] Mancel E (1999). Clinical aspects of ocular leptospirosis in New Caledonia (South Pacific). Aust N Z J Ophthalmol.

[20] Laing RW, TC, Toh CH (1990). Thrombotic thrombocytopenic purpura (TTP) complicating leptospirosis:a previously undescribed association. J Clin Pathol.

[21] Sukran K (2013). A leptospirosis case presenting with thrombotic thrombocytopenic purpura. Balkan Med J.

